# Supplementary figures and images for: Bayesian modeling of the impact of antibiotic resistance on the efficiency of MRSA decolonization
Source: PLoS Comput Biol. 2023 Oct 26;19(10):e1010898. doi: 10.1371/journal.pcbi.1010898 (PMC10629663; doi:10.1371/journal.pcbi.1010898)

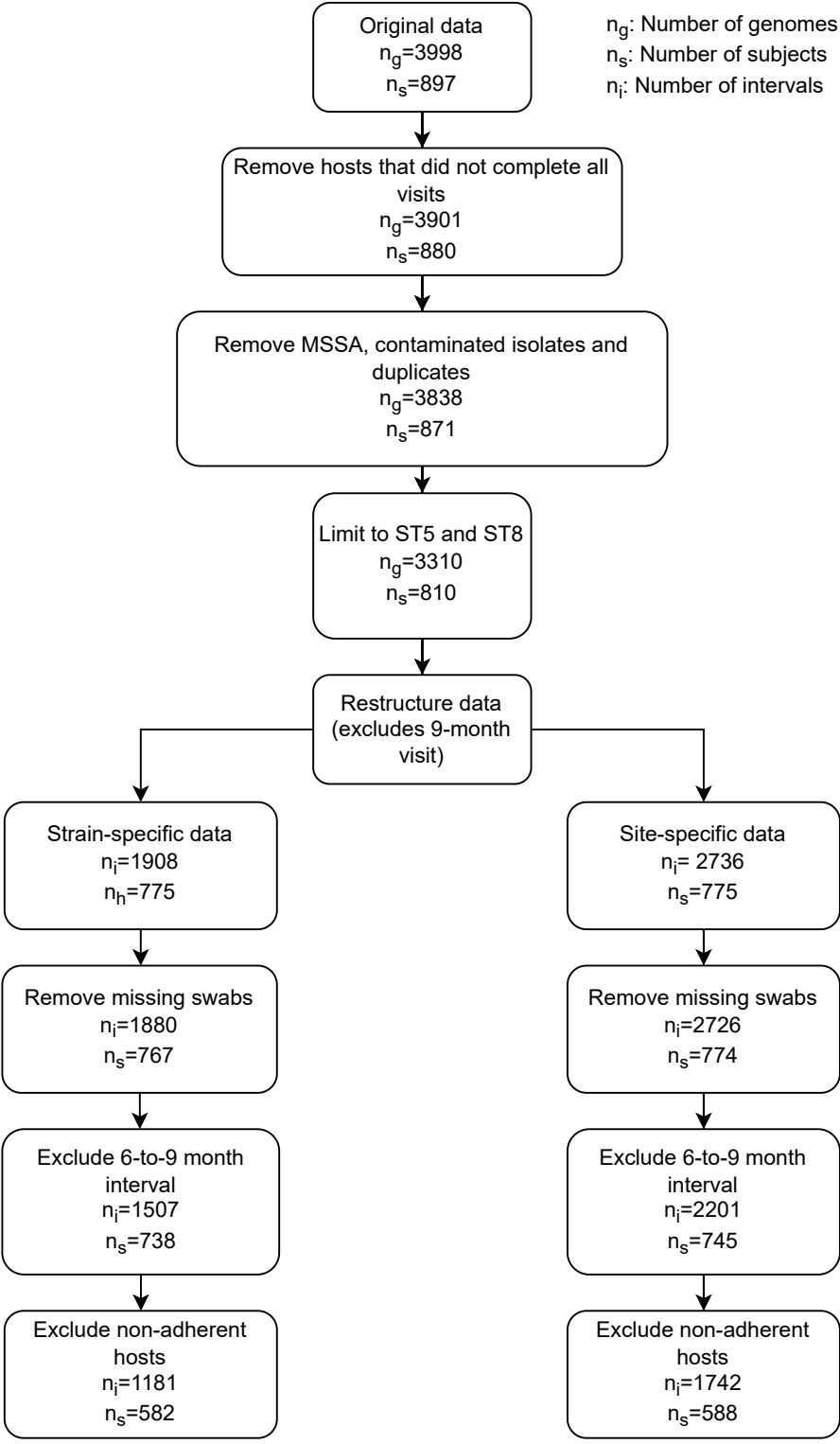

Supplement: S1 Appendix — Preprocessing steps to format the data for the survival analysis. ng represents the number of genomes (isolates), ni the number of intervals and ns the number of study subjects with sequencing data for the colonizing strains available. The number of observations is smaller after “Restructure data”, because the survival data are considered by interval: recruitment to 1-month, 1-month to 3-month and 3-month to 6-month. For example, in the original data one body site could have an isolate at each of the visits, contributing in total five observations in the original data. However, we only have three intervals to consider in the case of survival data, because we are interested in the clearance status of an MRSA strain between or at the end interval of two consecutive isolates (excluding the 6-month to 9-month interval). (PDF) [file pcbi.1010898.s001.pdf]

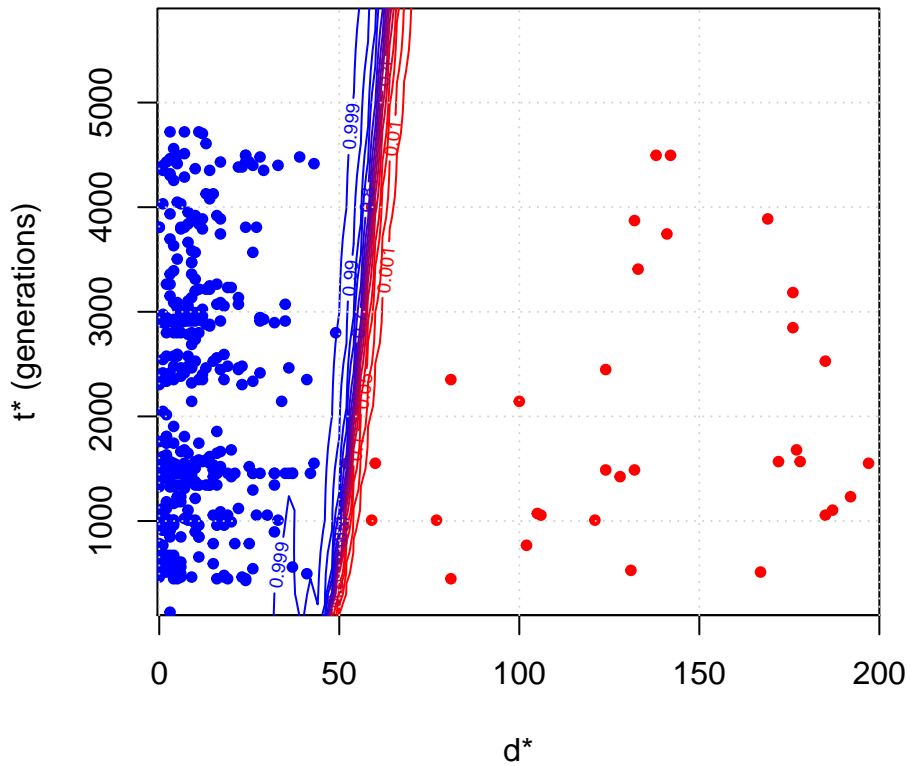

Supplement: S1 Fig — Contour plot detailing the same strain probability with SNP distance d* on the x-axis and the time between consecutive visits (in generations) on the y-axis. The probability of 0.5 was used to decide the threshold distance of 45 SNPs that was used to classify a pair of MRSA isolates observed in consecutive visits as the same or different strain. The BaeMBac software was run using 10 percent of randomly selected isolates from the education arm. The threshold was not sensitive to the amount of data, and the decolonization arm was not used as the BaeMBac software assumes a model of neutral evolution when calculating the same strain probability. (PDF) [file pcbi.1010898.s003.pdf]

# Impact of resistance on MRSA clearance, Strain Decolonization

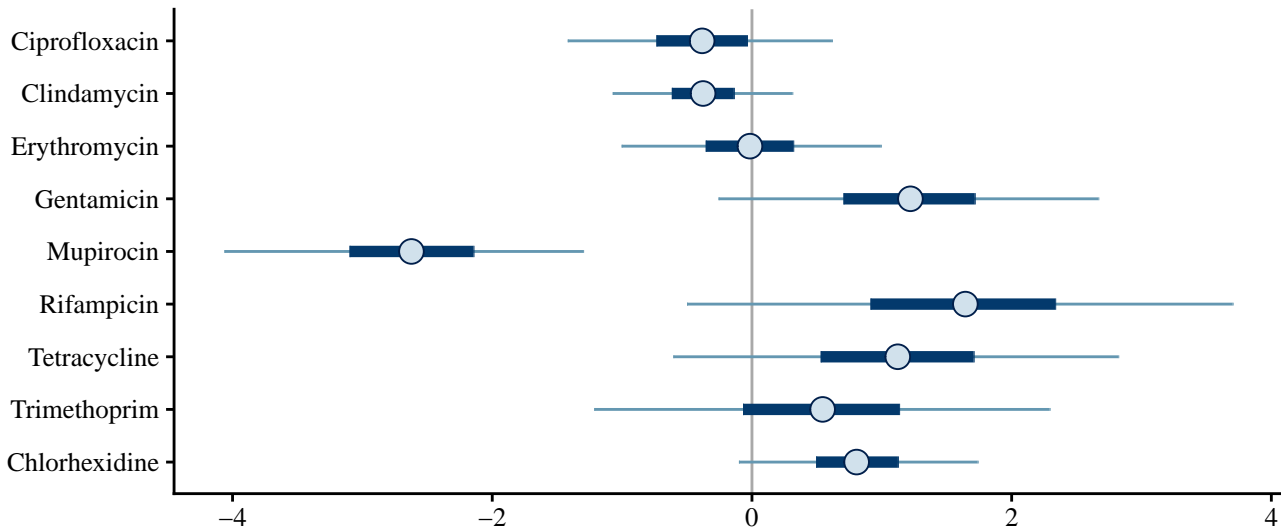

## Education

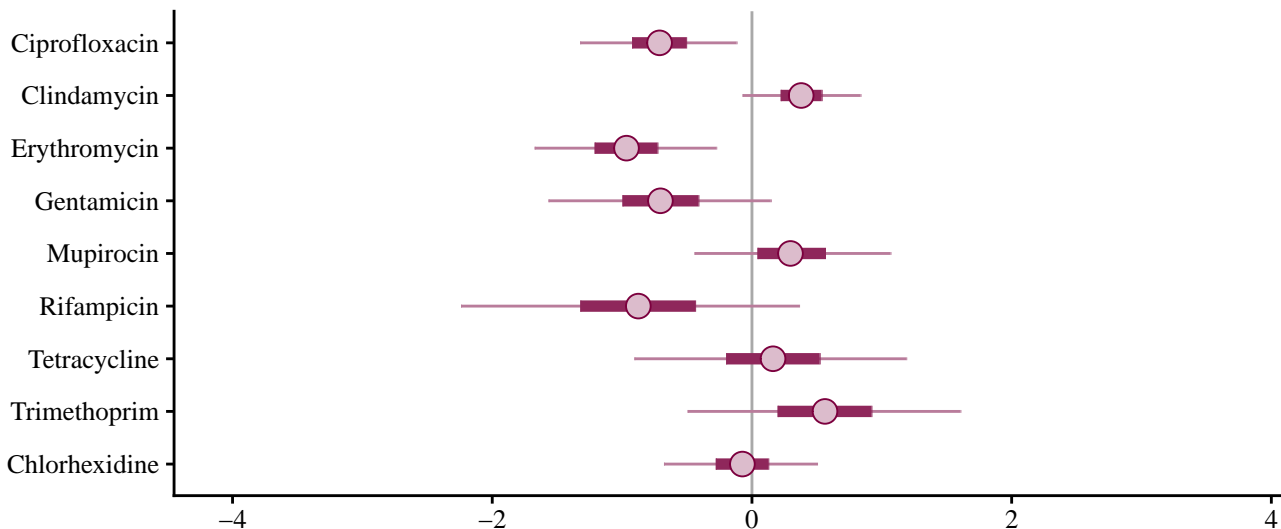

Supplement: S2 Fig — The 95% credible intervals for parameters β for all antibiotics with strain random effects included in the model, but excluding the subject-specific random effects. (PDF) [file pcbi.1010898.s004.pdf]

## Mupirocin HLR vs LLR, Host + Strain Decolonization

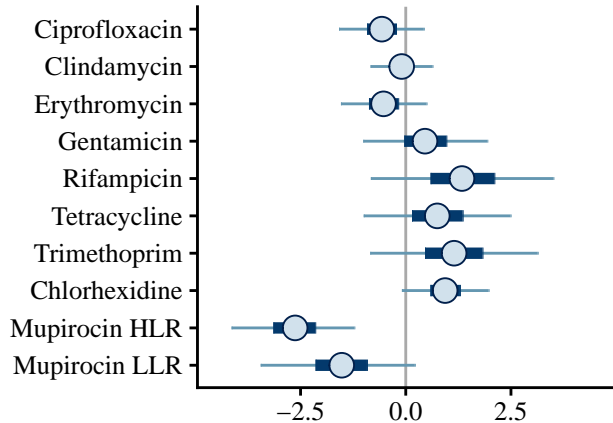

## Education

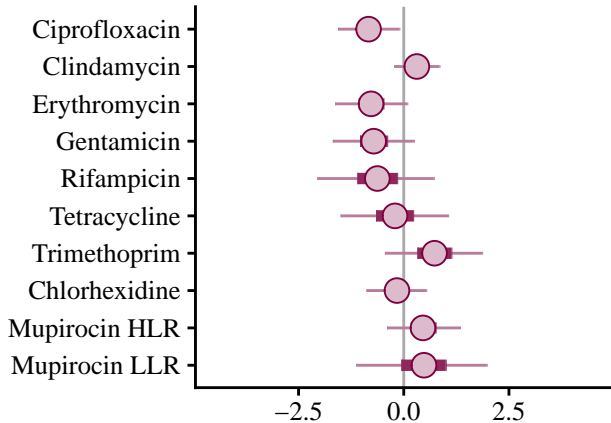

Supplement: S3 Fig — We included additional analysis of the phenotypic mupirocin resistance to complement our results on genetic resistance, and to separately consider low-level mupirocin resistance in our results. An MIC threshold of 512 ug/mL was used to distinguish between low-level and high-level resistance—a MIC value below 8 ug/mL indicated no resistance. Genetic resistance was used for other antimicrobials. The median and 50/95% credible intervals for each covariate are shown. In addition, a table comparing the resistance profiles of phenotypic and genotypic resistance for mupirocin and chlorhexidine is included. We can see that the results support the main analysis, with high-level mupirocin resistance contributing to persistence in the decolonization arm. We also note that low-level resistance is not significantly associated with persistence. Further studies could include characterization of low-level and high-level mupirocin resistance on a genetic level, for example by including a marker for the IleS gene. (PDF) [file pcbi.1010898.s005.pdf]

# Decolonization

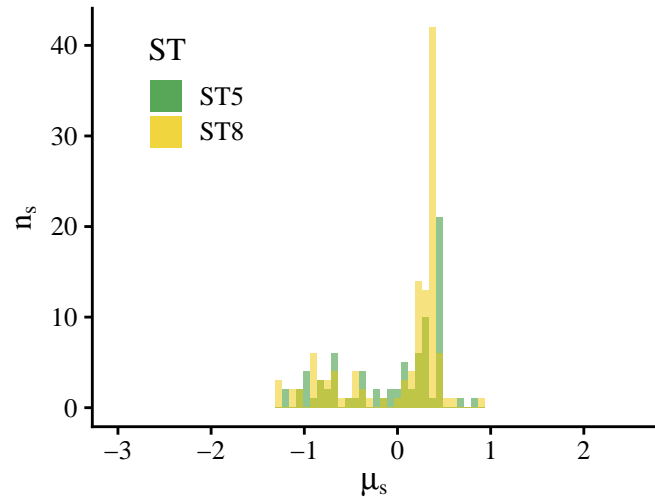

# Education

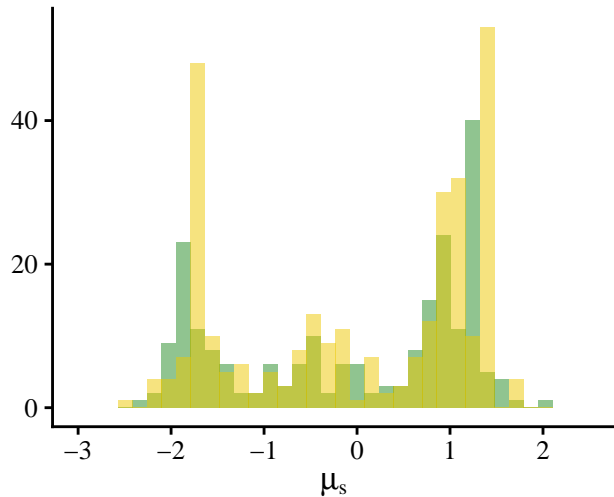

Supplement: S4 Fig — The strain-specific survival model was used to estimate the strain random effects. Histograms show the distributions of the strain random effect posterior means in the decolonization and education arms. (PDF) [file pcbi.1010898.s006.pdf]

# Decolonization

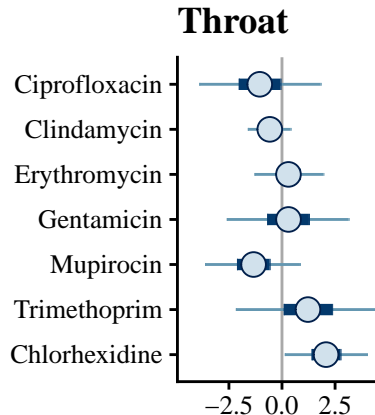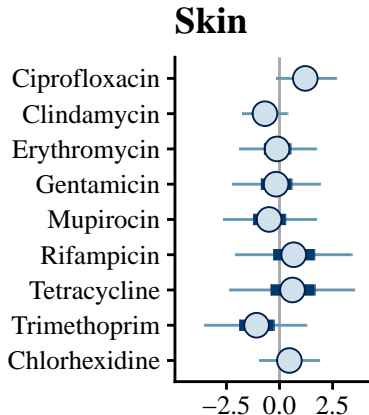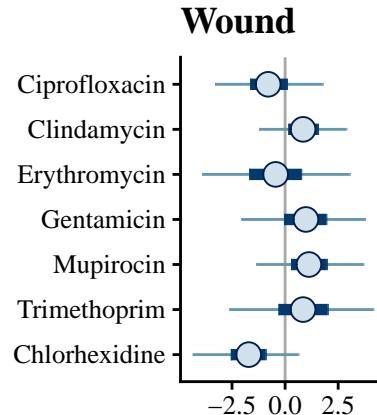

# Education

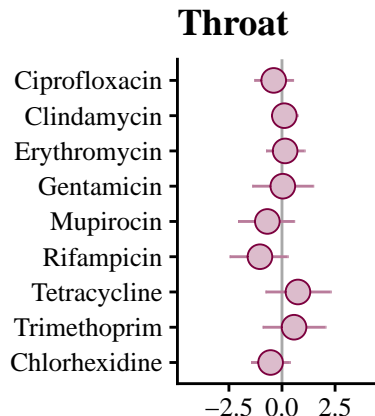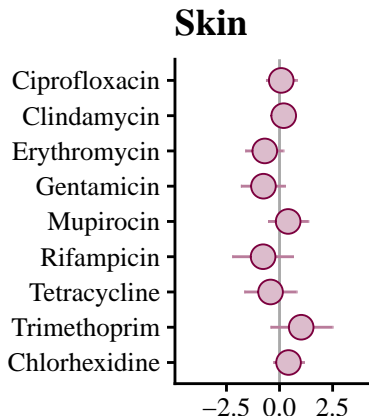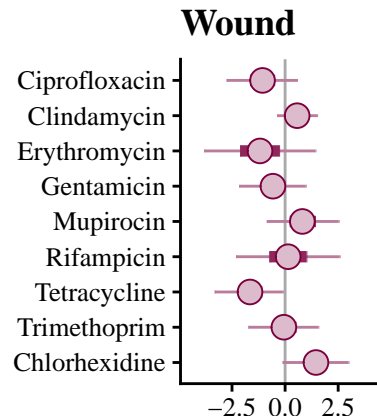

Supplement: S5 Fig — (PDF) [file pcbi.1010898.s007.pdf]

**Strain**

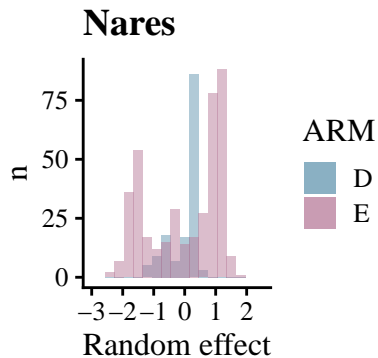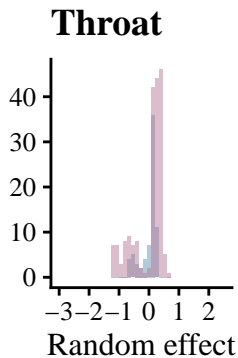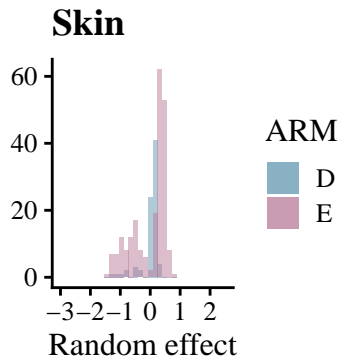

**Subject**

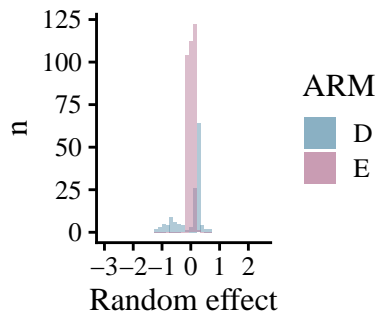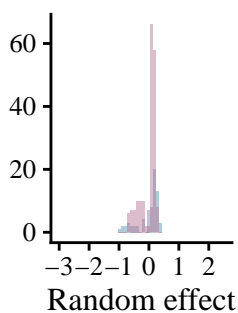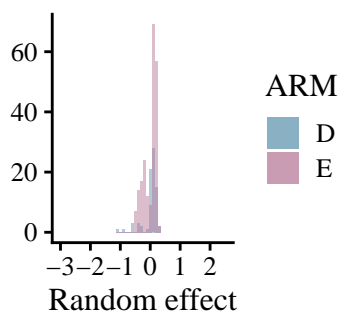

Supplement: S6 Fig — Strain random effects have more variation in the posterior means than study subject random effects, most notably in the nares. (PDF) [file pcbi.1010898.s008.pdf]

## Collinearity

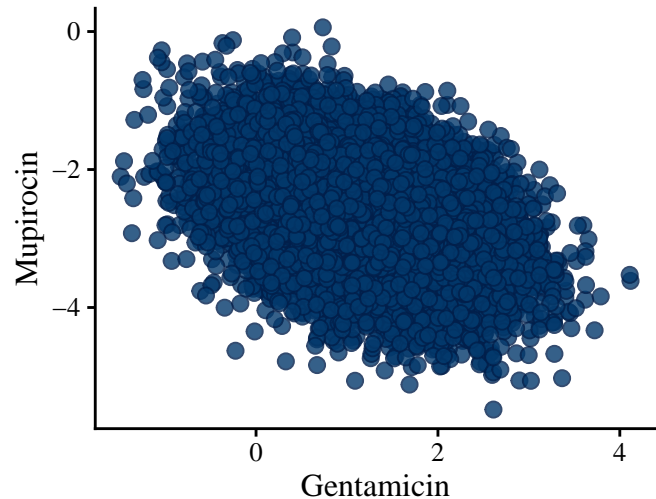

## Correlation

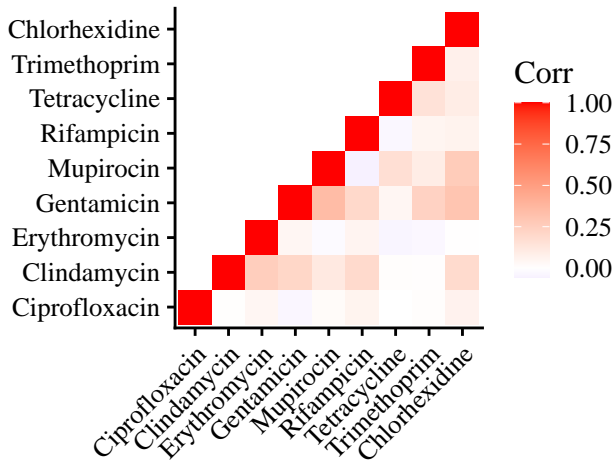

Supplement: S8 Fig — Collinearity represents the correlation between the effect sizes in the posterior samples for the given pair of antimicrobials, and it was visualized using the bayesplot [32] package. Furthermore, we included a heatmap of the Spearman correlation between each pair of antimicrobials. We see a small positive correlation between gentamicin and mupirocin resistance. Consequently, their effects are weakly negatively correlated in the posterior distribution. (PDF) [file pcbi.1010898.s010.pdf]

# Separate models for each covariate, Subject + Strain

## Decolonization

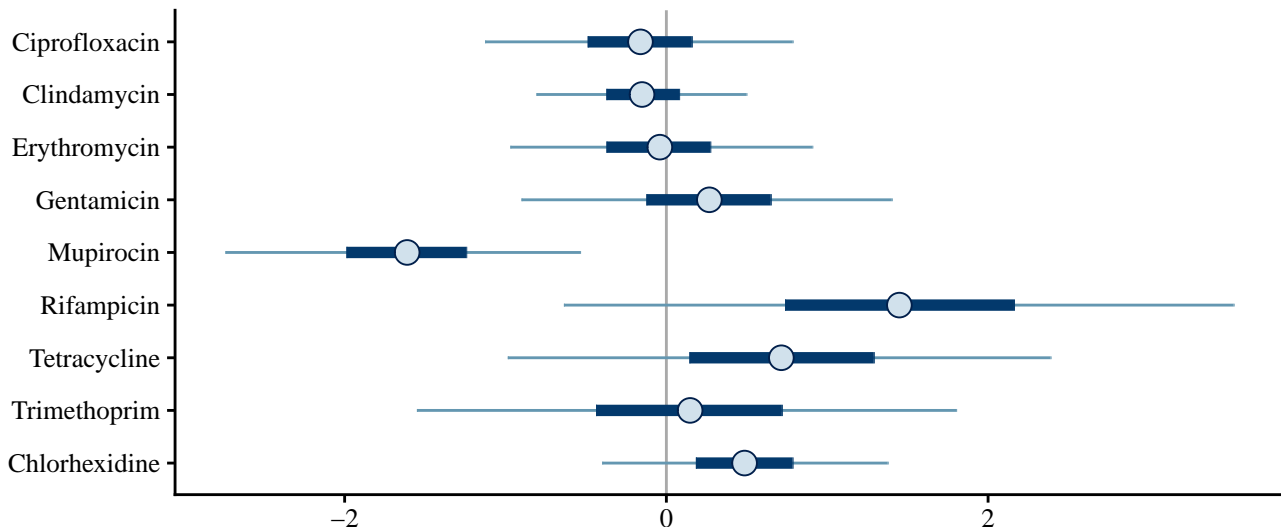

## Education

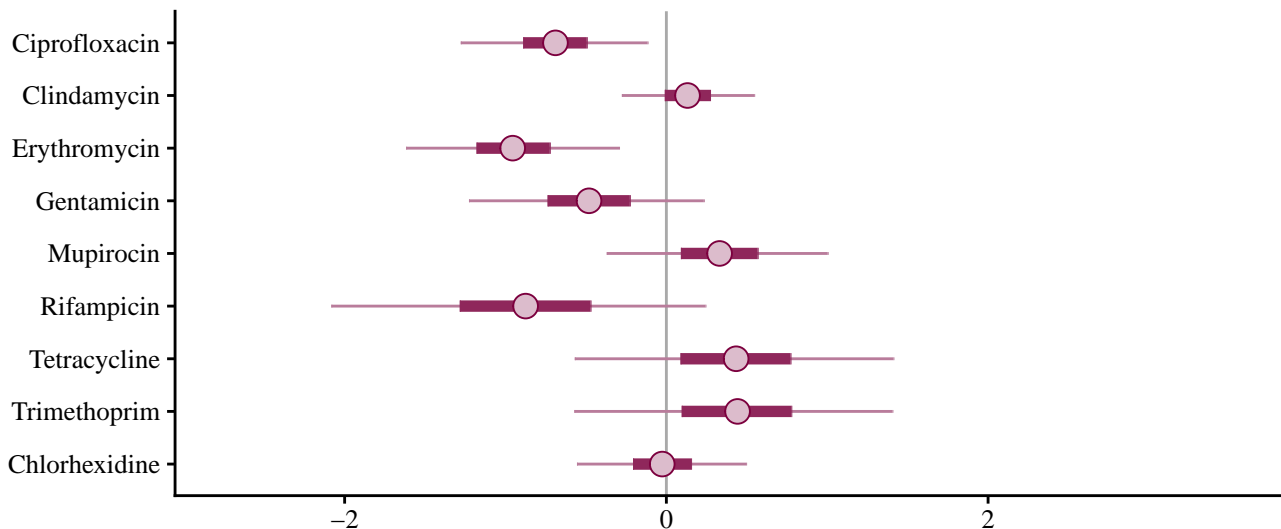

Supplement: S9 Fig — We repeated the Bayesian survival analysis for each antimicrobial in a separate model to account for the weak correlation between gentamicin and mupirocin (see S8 Fig). The figure shows the median and 50/95% CIs of the coefficient of each model. (PDF) [file pcbi.1010898.s011.pdf]
