# Supplementary material for: Bayesian modeling of the impact of antibiotic resistance on the efficiency of MRSA decolonization
Source: PLoS Comput Biol. 2023 Oct 26;19(10):e1010898. doi: 10.1371/journal.pcbi.1010898 (PMC10629663; doi:10.1371/journal.pcbi.1010898)
Supplement: S1 Table — Discrepancy between phenotypic and genetic resistance profiles in mupirocin and chlorhexidine. (PDF) [file pcbi.1010898.s002.pdf]

Mupirocin			
**Phenotypic**	**Genetic**		
	**Resistant**	**Susceptible**	**NA**
**High-level resistant**	216	30	10
**Low-level resistant**	0	51	1
**Susceptible**	30	2213	101
**NA**	28	529	52
**Chlorhexidine**			
**Phenotypic**	**Genetic**		
	**Resistant**	**Susceptible**	**NA**
**Reduced susceptibility**	43	236	35
**Susceptible**	235	1907	123
**NA**	91	527	45
