# Supplementary material for: Bayesian modeling of the impact of antibiotic resistance on the efficiency of MRSA decolonization
Source: PLoS Comput Biol. 2023 Oct 26;19(10):e1010898. doi: 10.1371/journal.pcbi.1010898 (PMC10629663; doi:10.1371/journal.pcbi.1010898)
Supplement: S7 Fig — In addition to the Bayesian survival model, we considered the standard Cox proportional hazards (PH) model with the antimicrobials as covariates. As another comparison, we included Bayesian logistic regression, conducted using the rstanarm package. Logistic regression does not consider the time difference between consecutive observations or censoring. Further, each of these is applied without the random effects. The figure shows 95% CIs for the coefficients of each antimicrobial. We see that qualitatively the results are similar to our model that includes the random effects, which highlights the robustness of our results. However, our model allows estimation of the host and strain specific contributions using the random effects, and leads to larger effect estimates for resistance types, which could be caused by the fact that effects are easier to estimate when additional noise due to random effects is first explained away. (PDF) [file pcbi.1010898.s009.pdf]

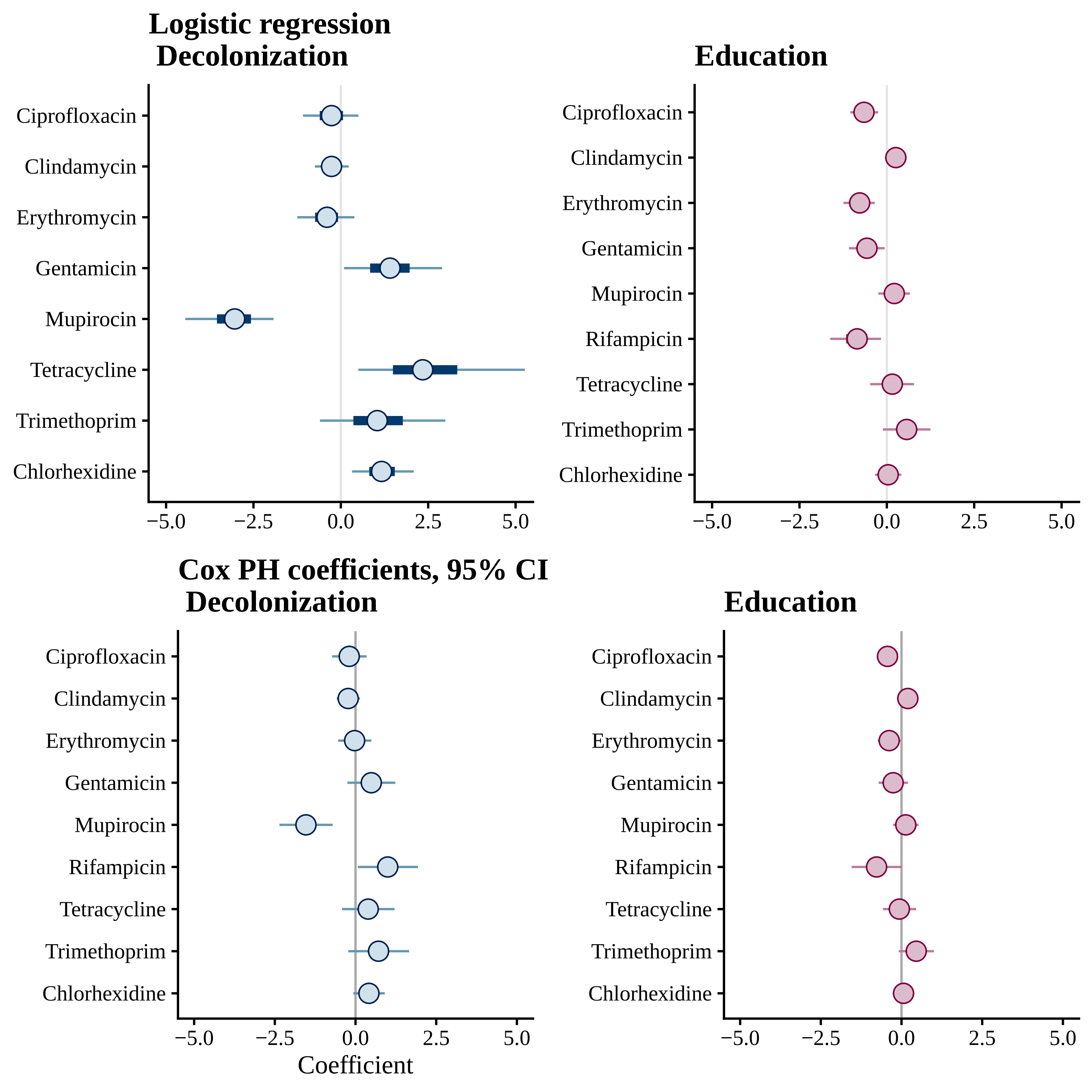

Logistic regression
Decolonization
Education
Ciprofloxacin
Clindamycin
Erythromycin
Gentamicin
Mupirocin
Rifampicin
Tetracycline
Trimethoprim
Chlorhexidine
−5.0
−2.5
0.0
2.5
5.0
Cox PH coefficients, 95% CI
Decolonization
Education
Coefficient
